# Placing the Responsibility

**Published:** 1920-12-18

**Authors:** 


### THE PUBLIC WELL-BEING.

### Placing the Responsibility.

### A PROBLEM AND A SOLUTION.

We desire to commend to readers of this section of The Hospital the appeal put forward with so much force in our leading columns. The object consistently aimed at in this section is to interest and assist the rapidly growing number of individuals and organisations in Great Britain devoted to the cause of public health; and to keep a still larger public in touch with the needs of the nation in the perpetual struggle against disease, suffering, and avoidably bad conditions of life, so that it may become more generally realised that this vitally important matter is not the concern merely of the Government and a comparatively select band of disinterested experts, but the concern of all.

Now it must be by this time fairly obvious that the various attempts that are being made in different directions to safeguard the public health, and to create an A1 population, cannot be expected to succeed unless the great network of voluntary hospitals, which extends from one end of the country to the other, is maintained in efficiency and developed in accordance with the growing needs of the people and the advancing demands of modern science. It is easy to see that without the hospitals the fight against disease would be reduced to a forlorn hope. They are the crux of the situation. Here it is that suffering humanity is treated, relieved, and cured with practically no cost to the individual, with the best medical and nursing skill, the latest apparatus, and with traditional and unwearying sympathy. Here it is that the young doctor is trained in knowledge and experience, and that opportunities are provided for carrying on those methods of research by which it is hoped finally to conquer the worst enemies of physical well-being. In face of these admitted facts, which we have grown accustomed to accept too much as a matter of course, it is surely a national reproach that at this moment the entire hospital system is passing through a serious crisis which threatens its collapse under the old conditions, or at least a severe curtailment of the privileges which it extends to all classes of people.

Even allowing for the greatly diminished wealth of Britain, and the intensified problem of living, this does not relieve any citizen in the kingdom from the moral obligation of keeping the hospitals going; for as they are a national insurance of the first importance against sickness in the community, their freedom from financial anxiety should be regarded as a first charge upon the community. We believe this responsibility is at last beginning to make itself felt, particularly among those who in the past have remained outside the circle of regular givers who, according to their means, have made sacrifices in order to pay tribute to the work of the hospitals. Christmas provides a suitable opportunity for pressing home this newly awakened sense of responsibility and stimulating it to active effort. We hope, therefore, that in every business house, in every factory, in every small shop, in every private home, an attempt will be organised to fill the empty coffers of the hospital committees; and that it will be no mere spasmodic effort, to disappear with the passing of the Christmas season, but will be converted in a regular and permanent scheme of contribution.

If every dividend drawer and wage-earner in the country developed the habit of giving twopence or threepence a week to the hospitals as regularly as they draw the dividend or the wage, there would be practically no hospital problem left to solve.

